# Clinical and Laboratory Features and Treatment Outcomes of Dengue Fever in Pediatric Cases

**DOI:** 10.7759/cureus.75840

**Published:** 2024-12-16

**Authors:** Nadia Nusrat, Kona Chowdhury, Susmita Sinha, Miral Mehta, Santosh Kumar, Mainul Haque

**Affiliations:** 1 Department of Pediatrics, Delta Medical College and Hospital, Dhaka, BGD; 2 Department of Pediatrics, Enam Medical College and Hospital, Dhaka, BGD; 3 Department of Physiology, Enam Medical College and Hospital, Dhaka, BGD; 4 Department of Pedodontics and Preventive Dentistry, Karnavati School of Dentistry, Karnavati University, Gandhinagar, IND; 5 Department of Periodontology and Implantology, Karnavati School of Dentistry, Karnavati University, Gandhinagar, IND; 6 Department of Pharmacology and Therapeutics, National Defence University of Malaysia, Kuala Lumpur, MYS; 7 Department of Research, Karnavati School of Dentistry, Karnavati University, Gandhinagar, IND

**Keywords:** aedes mosquito, breakbone fever, clinical attribute, dengue virus, febrile illness, high body temperature, hospitalized pediatric cases, laboratory findings, low- and middle-income countries (lmics), therapeutic intervention and consequence

## Abstract

Background

Globally, dengue fever (DF) is the leading cause of arthropod-borne viral illness, which considerably contributes to an atrocious death rate. The disease is now endemic in some parts of the world, including Bangladesh. The disorder exhibits a wide range of clinical and laboratory features in children. Judicial fluid resuscitation during the critical phase and prompt referral to the appropriate health facility can be lifesaving.

Objectives

This research appraised clinical and laboratory features and treatment outcomes of DF in pediatric cases.

Methods

This prospective investigative work was conducted at Islami Bank Hospital, Dhaka, India, from July to October 2023. The study included 135 admitted pediatric cases of DF, either dengue nonstructural protein 1 (NS1) or anti-dengue antibody IgM or IgG positive.

Results

Among the selected cases, boys were more predominant than girls, and most patients were in the age group of 5 to 10 years (n=46, 34%), most of them belonging to lower-middle-class families (n=56, 41.5%). All of the study participants had raised body temperatures, and most had abdominal pain (n=82, 60.7%), vomiting (n=77, 57%), cough (n=43, 31.9%), headache (n=38, 28.2%), body aches (n=32, 23.7%), and diarrhea (n=23, 17%). Dengue NS1 was positive in 91.1% (n=123) of cases. Raised hematocrit was found in 36.3% (n=49) of cases, leukopenia in 47% (n=63), and thrombocytopenia in 69.6% (n=94) of cases. Most of our patients were categorized as having DF (68.1%, n=92), followed by dengue with warning signs (16.3%, n=22), and severe dengue was present in 15.6% (n=21) of patients. Most were treated with crystalloid, and some with crystalloid and colloid solution. Fortunately, most of them recovered with no death.

Conclusion

DF may manifest with varied clinical and laboratory features in children. Appropriate treatment of critical phases, depending on clinical and laboratory features, is crucial to reducing dengue-induced miseries and fatal clinical outcomes among the pediatric population.

## Introduction

Dengue fever (DF) is the most critical illness among arthropod-borne viral diseases. It is transmitted by the Aedes mosquito, which is usually prevalent in tropical and subtropical areas. According to WHO, dengue has become endemic in more than 100 countries globally, comprising WHO regions of the Americas, Africa, Southeast Asia, the Eastern Mediterranean, and the Western Pacific. Cases are also increasing in the Eastern Mediterranean, Europe, and South American zones [1,2]. Multiple factors are responsible for the escalation of cases. Vectors are inhabiting countries where dengue was ingenuous previously. High temperature, humidity, and increased rainfall due to climate change and the effect of El Niño phenomena have significantly contributed to elevating the disease burden [2-5]. Moreover, delicate health systems, rapid urbanization, and migration of people across countries are also responsible [2,3].

The world experienced the highest number of dengue cases in 2023, penetrating over 80 countries. In the WHO region of America, 2300 people died among 4.5 million reported cases. The number of patients affected by dengue has also risen in Asian countries. Vietnam (36,900), Bangladesh (321,000), Thailand (150,000), and Malaysia (111,400) were affected chiefly [2]. According to the Directorate General of Health Services of Bangladesh, 1705 people had to embrace death due to this deadly viral infection [6]. United Nations International Children's Emergency Fund (UNICEF) reported that one child was affected for every five dengue cases, and for every six deaths, one belonged to the pediatric age group in Bangladesh [7]. Children pose a higher risk of developing severe dengue [8].

Four serotypes of dengue virus (DENV 1-4) persist in the environment, among which DEN-2 and DEN-3 are the dominant ones that make the infected persons symptomatic, causing significant morbidity and mortality [9-11]. In 2022 and 2023, Bangladesh experienced infection by DEN-2 and DEN-3 variants, causing havoc to our health system [12-14]. The virus enters the host by biting an infected Aedes mosquito through the skin [15].

The pathogenesis of dengue infection and its progression are not yet apprehended precisely [16]. A composite interaction between the host immune system, long noncoding RNAs, host genes, and virulence factor of DENV plays a pivotal role in the progression and complication of the disease [17]. Furthermore, DENV can infect various immune cells, including macrophages, monocytes, mast cells, dendritic cells, and B and T cells, further disrupting the antiviral capacity of these cells and spreading the virus [18]. Despite the infectious nature of all serotypes of DENV, primary infection with one serotype often remains asymptomatic or may present as an uncomplicated febrile illness [19]. After DENV primary infection, immunological memory cells are produced against that specific serotype. Consecutive infection with different serotypes causes the pre-existing immunoglobulins to bind with the heterologous serotype. This antigen-antibody complex is unable to neutralize the virus. On the contrary, it escalates infection [20] and may present as a severe life-threatening condition [21].

DENV affects various body organs, so a wide range of diversity is observed in clinical manifestations [22]. The usual symptoms in children include the sudden appearance of high fever, retro-orbital pain, severe headache, joint and muscle pain, vomiting, nausea, and swollen glands, which may be mistaken for diseases like malaria, chikungunya, or Zika [23]. The Bangladesh government has constructed a guideline for the clinical management of dengue cases. They described the illness as dengue syndrome, which encompasses undifferentiated DF, dengue hemorrhagic fever (DHF), and expanded dengue syndrome (EDS). DF is marked as DHF when there is evidence of plasma leakage during the critical phase. When DHF presents with evidence of circulatory failure, it is marked as dengue shock syndrome (DSS). There is a rare manifestation of DF when the brain, liver, kidney, or heart are involved with or without plasma leakage, known as EDS. EDS usually occurs in the presence of comorbidities or coinfections [24] and significantly increases mortality risk [25].

Laboratory confirmation of dengue virus infection is crucial as it can be easily misdiagnosed with other viral diseases, and diagnostic delay may cause life-threatening conditions [26]. Traditional tests like reverse transcription polymerase chain reaction (RT-PCR), polymerase chain reaction (PCR), or enzyme-linked immunosorbent assay (ELISA) are expensive, time-consuming, and need expert technical support [27]. Rapid diagnostic tests are currently used to identify dengue nonstructural protein 1 (NS1) antigen, IgG, and IgM antibodies for diagnostic purposes [28]. Usually, early diagnosis of dengue is based on detecting NS1 antigen in the blood [29], as the IgM antibody can only be detected after the fifth or sixth day of the clinical manifestation of the illness [30].

After viral infection, NS1, a glycoprotein in flavivirus, is secreted from affected cells within hours, so detecting NS1 in blood has become an excellent alternative to RT-PCR. This method has gained better acceptance among healthcare providers in low- and middle-income countries (LMICs) as it is less time-consuming, cheaper, and can be done at the point of care [31]. Some studies have reported a correlation between elevated NS1 protein levels and hemorrhagic dengue cases [32-34], while few studies differed with this [35]. NS1 detection by the rapid diagnostic test (RDT) method showed 76.5% sensitivity, but when ultrafiltration was used to concentrate the sera three times before testing, the sensitivity reached 80.4% and showed 100% specificity [36]. IgM antibody against dengue becomes detectable after five days of infection and may persist for 1-3 months. IgG appears later, usually in the 9th or 10th day of fever, and may be detectable in the lower titer for decades [24]. Detection of dengue IgM/IgG by rapid immunochromatographic test (ICT) is a valuable alternative in a resource-limited area, as standard ELISA requires money, time, and a well-equipped lab [37]. One study from Senegal concluded that RDT NS1 antigen, IgM, and IgG detection could be a practical substitute test in point-of-care, especially during an outbreak [38].

Blood counts often show leucopenia and thrombocytopenia [39-41], and a reduced ratio of neutrophil to lymphocyte indicates a critical phase and initiation of plasma leakage [42]. A platelet count of less than 100,000/µL is typical [43,44]. Significant rise in hematocrit, reduced platelet count [44,45], hypoalbuminemia [46,47], and reduced prothrombin level [40,48-51] are associated with DHF and DSS. Other abnormalities include escalation of transaminase level, hyponatremia, and increased serum urea level [24,40,51,52]. Pleural effusion is typical in dengue patients with plasma leakage and is frequently diagnosed by chest X-ray [53,54]. Ultrasonography can be a helpful tool to detect minimal pleural effusion and ascites. Sonographic findings of thickening of the gallbladder indicate potential plasma leakage in severe dengue [55-57].

There is no definitive treatment plan. Thus, supportive management is the mainstay for dengue management [1,57-59]. Meticulous fluid replacement is the cornerstone of the treatment modality [60]. Crystalloid solutions are the initial fluid of choice, and they are sometimes replaced by colloids in DSS [61]. Transfusion of platelets prophylactically is discouraged as it has no beneficial effect [62]. Adequate bed rest and paracetamol are advised during the acute febrile phase [63,64]. Complications due to vital organ involvement should be meticulously addressed [58]. Diligent fluid reduction during the resorptive phase is critical to prevent hypervolemia. Disproportionate fluid administration may result in heart failure and pulmonary edema [8,65].

Globally, more considerable dengue outbreaks have been observed recently, with various serotypes spreading in non-endemic zones [66-69]. Moreover, under-reporting prevails as disease surveillance often fails to capture all affected cases. Health authorities often fail to realize dengue's factual scenario and atrocities [70]. Healthcare professionals still lack adequate knowledge and practice regarding dengue despite the colossal patient load [71-73], which endangers patient management. Medical practitioners should be aware of various manifestations of DF and the outcome and prognosis of the disease to improve health care quality. Hence, this study aimed to evaluate DF's clinical and laboratory features and treatment outcomes in children in admitted patients.

Objectives of this study

This study aims to investigate the clinical and laboratory manifestations, socio-demographic characteristics, and treatment outcomes of pediatric DF cases admitted to Islami Bank Hospital, Dhaka, India, from July to October 2023.

## Materials and methods

Study type, population, and sample size

It is a prospective study conducted at Islami Bank Hospital from July to October 2023. About 135 pediatric patients aged 0 to 18 were diagnosed with DF and admitted, including 135 patients admitted to the hospital during the earlier period.

Inclusion and exclusion criteria

Children aged 0-18 who have been diagnosed with DF (either dengue NS1 positive or anti-dengue antibody positive) are included in the study. The patient ′s parents or caregivers are excluded without written informed consent.

Sample size justification

It has been reported that from January 1 to August 7, 2023, the Ministry of Health and Family Welfare of Bangladesh reported 69,483 laboratory-confirmed dengue cases (WHO, Dengue-Bangladesh, 2024) [74]. The prevalence of dengue in Bangladesh as of June 2023 is approximately 0.040% or about 40 cases per 100,000 population. Considering this prevalence of dengue with a precision of 0.04% and a 95% confidence interval, the sample size was 92. Design effect was used for the sample size calculations as a correction factor to adjust the required sample size to avoid any unknown bias due to data collected from one community hospital in Dhaka, socioeconomic status (SES), and the high transmission rate of dengue at present. Thus, adding a design effect 1.5, the estimated sample was 138. We were able to enroll 135 participants for this study.

Operational definitions

The World Health Organization defined dengue as "a combination of ≥2 clinical findings in a febrile person who lives in or traveled to (in the last 14 days) a dengue-endemic area. Clinical findings include nausea, vomiting, rash, aches and pains, a positive tourniquet test, leukopenia, or any warning sign" [75].

Dengue with warning signs "include abdominal pain or tenderness, persistent vomiting, clinical fluid accumulation, mucosal bleeding, lethargy, restlessness, and liver enlargement" [75].

Severe dengue "is defined as dengue with any of the following clinical manifestations: severe plasma leakage leading to shock or fluid accumulation with respiratory distress; severe bleeding; or severe organ impairment such as hepatitis (elevated transaminases ≥1,000 IU/L), impaired consciousness, or heart impairment" [75].

Data collection

Appropriate history was taken, and all clinical and laboratory information was gathered in a predesigned spreadsheet.

Data analysis

Descriptive statistics were examined for outcome variables by child sex. As this was a descriptive study, overall distributions were assessed. To determine differences in outcomes by child sex, we used the chi-square test for binary variables and the chi-square test with Yates' continuity correction for ordinal variables. Associations between child sex and weight, fever duration, platelet count, and average hospital stay were evaluated using the independent sample t-test. Data processing and analysis were conducted in STATA (version 15), with significance set at a p-value of 0.05.

Ethical approval

**Figure 1:**
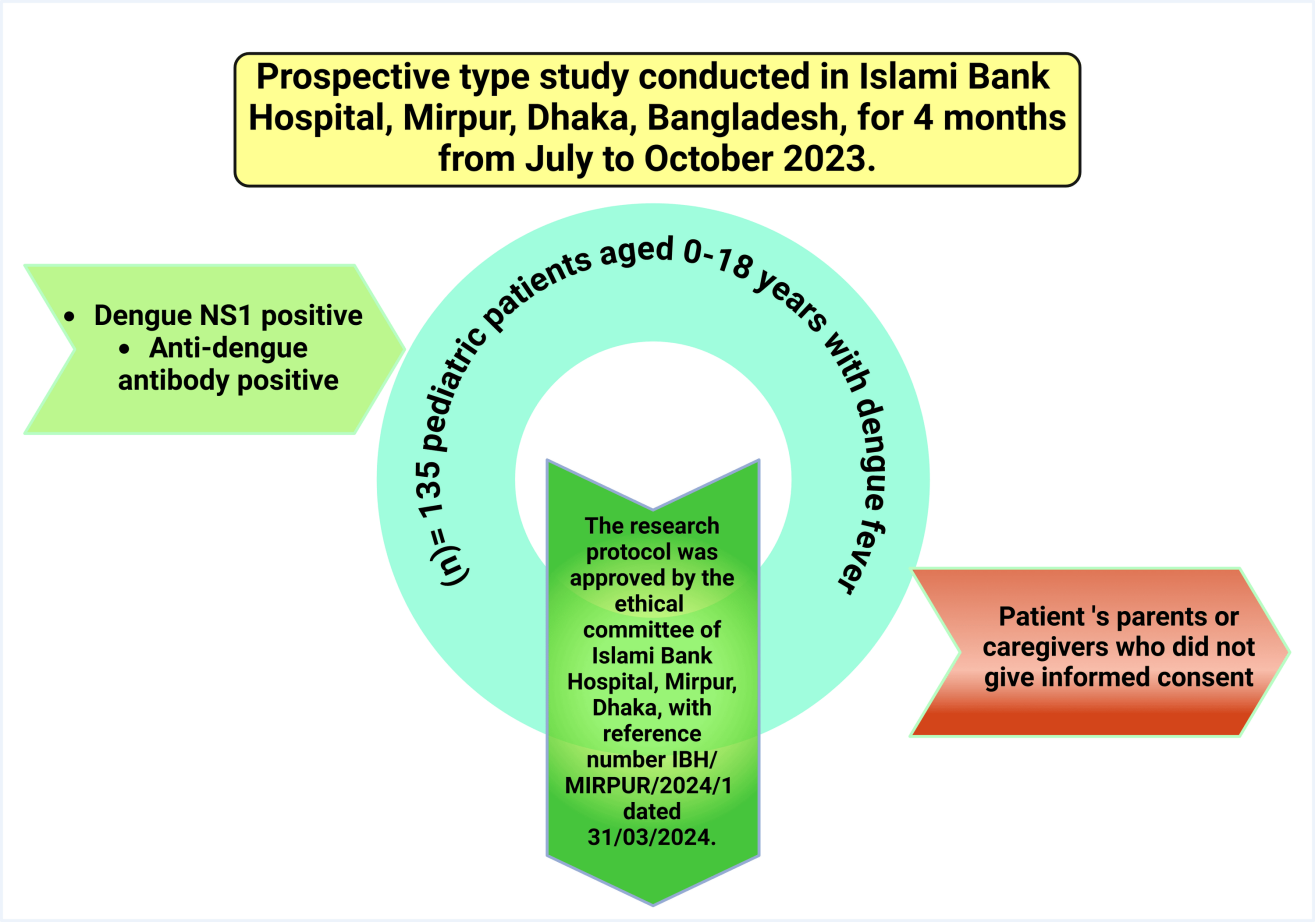
Illustration of the study methodology Note: This figure was drawn using the premium version of BioRender (https://BioRender.com/d36w685) [76] with agreement license number EM27ID6VNP. Illustration Credit: Susmita Sinha NS1: nonstructural protein 1

The Ethical Committee of Islami Bank Hospital, Dhaka, India, approved the research protocol with reference number IBH/MIRPUR/2024/1, dated March 31, 2024. Parents or caregivers of these research participants were given written informed consent and verbally explained about the study design, the purpose of the study, and their right to withdraw them from the project at any time for any reason. Figure 1 illustrates the methodology of this paper.

## Results

The demographic characteristics of children admitted with dengue were analyzed, and 135 cases were examined. Among them, 86 were boys and 49 were girls. The mean age of admitted children was 91.6 months, with boys slightly younger at 81.7 months compared to girls. The lowest age was three months in a boy. Most patients were in the age group of >5-10 years (34%, n=46). Age and sex-wise distribution of the patients was described in Table 1. SES distribution revealed that most cases were from the lower middle class (41.5%, n=56), followed by the upper middle class (n=41, 30.4%) and upper class (n=34, 25.2%). Most patients (n=120, 88.8%) were from the Mirpur area of Dhaka city, Bangladesh. Only 10 (7.4%) patients were from outside Mirpur and 5 (3.7%) from outside Dhaka, Bangladesh. Child weight varied with a mean of 27.1 kg, with boys slightly heavier at 28.9 kg compared to girls at 24.0 kg. Comorbidities were also observed, with obesity, overweight, underweight, and other conditions present in varying proportions among the admitted children. However, there were no statistically significant differences in age, SES, child weight, or comorbidities between boys and girls, indicating similar demographic profiles among the genders within the cohort (Table 2).

**Table 1:** Distribution of patients according to age and sex

Age in year	Overall (n=135)	Boys (n=86)	Girls (n=49)
<1 year	12 (8.9%)	7 (8.1%)	5 (10.2%)
1-5 years	37 (27.4%)	25 (29%)	12 (24.5%)
>5-10 years	46 (34%)	26 (30.2%)	20 (40.8%)
>10-15 years	30 (22.2%)	19 (22%)	11 (22.4%)
>15 years	10 (7.4%)	9 (10.5%)	1 (2%)

**Table 2:** Demographic characteristics of the admitted child with dengue Note: Data was presented as mean±SD for continuous variables and number with percent in the parenthesis for categorical variables. The chi-square test with Yates' continuity correction was applied to estimate the p-value. kg: kilogram *Others: asthma, heart diseases, nephrotic syndrome, thalassemia minor, hypothyroidism, cholelithiasis, and anxiety disorder.

Variables		Overall (n=135)	Boys (n=86)	Girls (n=49)	p-value
Age, in month		88.0±57.0	91.6±6.55	81.7±7.09	0.334
SES condition	Upper class	34 (25.2%)	21 (24.4%)	13 (26.5%)	0.817
	Upper middle class	41 (30.4%)	28 (32.6%)	13 (16.5%)	
	Lower middle class	56 (41.5%)	35 (40.7%)	21 (42.8%)	
	Lower class	4 (2.9%)	2 (2.3%)	2 (4%)	
Child weight, kg		27.1±17.2	28.9±19.0	24.0±13.2	0.113
Comorbidities	Obesity	14 (10.4%)	10 (11.6%)	4 (8.16%)	0.627
	Overweight	11 (8.15%)	5 (5.81%)	6 (12.2%)	
	Underweight	9 (6.67%)	5 (5.81%)	4 (8.16%)	
	Others*	8 (5.92%)	5 (5.81%)	3 (6.10%)	

Fever was present in all patients (n=135, 100%). Common symptoms included abdominal pain (n=82, 60.7%), vomiting (n=77, 57%), headache (n=38, 28.2%), and cough (n=43, 31.9%). Retroorbital pain was less prevalent, reported in only 0.74% (n=1) cases. Boys exhibited slightly higher rates of most symptoms than girls, although the differences were not substantial. Bleeding manifestations were observed in 5.93% (n=8) of cases, with boys' predominance. Epistaxis was the most common bleeding manifestation. Convulsions were reported in 2.96% (n=4) of cases (Table 3). The duration of fever averaged 3.44 days, with boys and girls experiencing similar durations at 3.50 days and 3.33 days, respectively. These findings provide insight into the clinical presentation of dengue among admitted patients, highlighting common symptoms and their distribution among sexes.

**Table 3:** Clinical symptoms of the dengue admitted patients Note: Data was presented as mean±SD for continuous variables and number with percent in the parenthesis for categorical variables. Chi-square for binary variables was used to estimate the p-value, and an Independent sample t-test for continuous variables was used to estimate the p-value.

Variables	Overall (n=135)	Boys (n=86)	Girls (n=49)	p-value
Clinical symptoms				
Headache	38 (28.2%)	27 (31.4%)	11 (22.5%)	0.266
Body ache	32 (23.7%)	21 (24.4%)	11 (22.5%)	0.796
Retro orbital pain	1 (0.74%)	1 (1.16%)	0	0.449
Abdominal pain	82 (60.7%)	56 (65.1%)	26 (53.1%)	0.168
Vomiting	77 (57.0%)	51 (59.3%)	26 (53.1%)	0.481
Loose stool	23 (17.0%)	20 (23.3%)	3 (6.12%)	0.011
Bleeding manifestation	8 (5.93%)	7 (8.14%)	1 (2.04%)	0.214
Convulsion	4 (2.96%)	2 (2.33%)	2 (4.08%)	0.967
Cough	43 (31.9%)	28 (32.6%)	15 (30.6%)	0.815
Rash	16 (11.9%)	11 (12.8%)	5 (10.2%)	0.808
Duration of fever, days	3.44±1.17	3.50±1.16	3.33±1.20	0.520

Hypotension or narrow pulse pressure (narrow PP) was observed in 11.9% (n=16) of patients, while 3.7% (n=5) had non-recordable blood pressure readings. Regarding plasma leakage signs, isolated pleural effusion was rare, reported in only 0.74% (n=1) of cases, and isolated ascites in 1.48% (n=2). Thirty-six cases (26.7%) exhibited pleural effusion and ascites, while 71.1% (n=96) showed no plasma leakage. Most patients had minimal ascites; nine had moderate ascites with bilateral moderate pleural effusion. Unilateral (right) pleural effusion and bilateral effusion were almost equal. Bulged fontanelle, a potential sign of increased intracranial pressure, was present in 1.48% (n=2) of cases and infants. The gender-wise comparison indicates similar distributions of blood pressure categories and plasma leakage signs between boys and girls, suggesting no significant differences in these clinical parameters based on sex (Table 4).

**Table 4:** Gender comparison of blood pressure and plasma leakage in pediatric dengue patients Note: Data was presented as numbers with percentages in the parenthesis. For 2×2 contingency tables, we used the chi-square test, and for 2×3 contingency tables, the chi-square test with Yates' continuity correction was applied to estimate the p-value. PP: pulse pressure

Variables		Overall (n=135)	Boys (n=86)	Girls (n=49)	p-value
Blood pressure	Normal	114 (84.4%)	72 (83.7%)	42 (85.7%)	0.872
	Hypotensive/narrow PP	16 (11.9%)	11 (12.8%)	5 (10.2%)	
	Non-recordable	5 (3.70%)	3 (3.49%)	2 (4.08%)	
Sign plasma leakage	Pleural effusion	1 (0.74%)	1 (1.16%)	0	0.384
	Ascites	2 (1.48%)	2 (2.33%)	0	
	Both	36 (26.7%)	24 (27.9%)	12 (24.5%)	
	No	96 (71.1%)	59 (68.6%)	37 (75.5%)	
Bulged fontanelle		2 (1.48%)	1 (1.16%)	1 (2.04%)	0.690

The majority tested positive for the dengue NS1 test, with 91.1% (n=123) overall positivity. Most patients (n=77, 57%) had standard hematocrit values, and 36.3% (n=49) of patients had raised hematocrit values, of which eight patients (n=8, 5.9%) had more than thirty percent rise. A small percentage (n=9, 6.7%) had falling hematocrit. Leukopenia was observed in 47% (n=63) of cases. Most patients (n=64, 47.4%) had an average WBC count, and only a tiny percentage (n=8, 5.9%) had leukocytosis. The majority (n=93, 68.9%) showed increased lymphocytes concerning neutrophil count (N<L). Thrombocytopenia was present in most patients (n=94, 69.6%). Among them, 20.2% (n=19) had severe thrombocytopenia (<20,000/cumm). Platelet counts varied, with a median of 49,500 (IQR: 24,000-90,000), slightly lower among girls than boys. Serum SGPT was raised in 25 patients (18.6%); three significantly rose among them. Most patients (n=101, 74.8%) had average serum albumin levels, and 25.2% (n=34) showed hypoalbuminemia. Electrolyte abnormality was observed in only 4.4% (n=6) of cases; hyponatremia was an essential finding among them. Most patients had routine ultrasound (USG) abdomen and chest X-ray results, with 68.9% (n=93) and 71.1% (n=96) showing normal findings, respectively (Table 5). In most cases, USG abnormalities were ascites and pleural effusion. Five cases showed hepatomegaly, two had both liver and spleen enlargement, one had only splenomegaly, and two had a finding of acalculous cholecystitis. Chest X-ray abnormalities were evidence of pleural effusion and, in some cases, features of secondary infection (pneumonia) (Table 6).

**Table 5:** Laboratory findings of patients Note: Data was presented as mean±SD for continuous variables and number with percent in the parenthesis for categorical variables. For 2×2 contingency tables, the chi-square test was used. For 2×3 contingency tables, the chi-square test with Yates' continuity correction was applied to estimate the p-value. An independent sample t-test for continuous variables was used to assess the p-value. IQR: interquartile range; SGPT: serum glutamate pyruvate transaminase

Variables		Overall (n=135)	Boys (n=86)	Girls (n=49)		p-value
Dengue test (NS1) positive		123 (91.1%)	77 (89.5%)	46 (93.9%)		0.394
Dengue antibody positive		13 (9.63%)	10 (11.6%)	3 (6.12%)		0.297
Hematocrit	Normal	77 (57%)	49 (57%)	28 (57.1%)		0.600
	Raised	49 (36.3%)	30 (35%)	19 (38.8%)		
	Fall	9 (6.7%)	7 (8.1%)	2 (4%)		
Total count of WBC	Normal	64 (47.4%)	41 (47.7%)	23 (47%)		0.877
	Leukopenia	63 (47%)	38 (44.2%)	25 (51.0%)		
	Leukocytosis	8 (5.9%)	7 (8.1%)	1 (2.1%)		
Differential count of WBC	Neutrophil < Lymphocyte	93 (68.9%)	59 (68.6%)	34 (69.3%)		0.911
	Neutrophil > lymphocyte	42 (31.1%)	27 (31.4%)	15 (30.6%)		
Platelets count, median (IQR)	49,500 (24,000, 90,000)	55,000 (25,000, 90,000)	45,000 (21,000, 80,000)	Platelets count, median (IQR)		0.374
Cut platelets count		n=94	n=63	n=31		
	<20,000	19 (20.2%)	12 (19.0%)	7 (22.6%)		0.821
	20,000-50,000	29 (30.9%)	18 (28.6%)	11 (35.5%)		
	50,000-100,000	28 (29.8%)	20 (31.7%)	8 (25.8%)		
	100,000-150,000	18 (19.1%)	13 (20.6%)	5 (16.1%)		
Serum SGPT	Normal	110 (81.5%)	69 (80.2%)	41 (83.7%)		0.678
	Increased	25 (18.6%)	17 (19.7%)	8 (16.35)		
Serum albumin	Normal	101 (74.8%)	64 (74.4%)	37 (75.5%)		0.857
	Reduced	34 (25.2%)	22 (25.6%)	12 (24.5%)		
Serum electrolytes	Normal	129 (95.6%)	83 (96.5%)	46 (93.9%)		0.782
	Abnormal	6 (4.4%)	3 (3.5%)	3 (6.1%)		
Ultrasonography abdomen	Normal	93 (68.9%)	58 (67.4%)	35 (71.4%)		0.620
	Abnormal	42 (31.1%)	28 (32.6%)	14 (28.6%)		
Chest X-ray	Normal	96 (71.1%)	60 (69.8%)	36 (73.5%)		0820
	Abnormal	39 (28.9%)	26 (30.2%)	13 (26.5%)		

**Table 6:** Treatment of dengue patients Note: Data was presented as numbers with percentages in the parenthesis. Chi-square was used to estimate the p-value.

Treatment	Overall (n=135)	Boys (n=86)	Girls (n=49)	p-value
ORS	135 (100%)	86 (100%)	49 (100%)	0.999
Crystalloid	115 (85.2%)	74 (86.1%)	41 (83.7%)	0.699
Crystalloid with plasmasol	12 (8.89%)	8 (9.30%)	4 (8.16%)	0.211
Crystalloid with albumin	8 (5.93%)	4 (4.65%)	4 (8.16%)	0.999
Blood transfusion	12 (8.89%)	10 (11.6%)	2 (4.08%)	0.050
Use of antibiotic	21 (15.6%)	15 (17.4%)	6 (12.2%)	0.423

Patients were categorized into groups A: DF, B: dengue with warning signs, and C: severe dengue. Most patients (n=92, 68.1%) were in group A, followed by dengue with warning signs (n=22, 16.3%). Fortunately, most patients (n=131, 97.0%) recovered, with a median duration of hospital stay of 3.73 days. A few patients (n=4, 2.96%) were referred to the pediatric intensive care unit (PICU) for better management. These findings underscore the prevalence of dengue, with the majority experiencing positive outcomes and relatively short hospital stays (Table 7).

**Table 7:** Outcome of dengue patients Note: Data was presented as mean±SD for continuous variables and number with percent in the parenthesis for categorical variables. For 2×2 contingency tables, we used the chi-square test. For 2×3 contingency tables, the chi-square test with Yates' continuity correction was applied to estimate the p-value. An independent sample t-test for continuous variables was used to assess the p-value. PICU: pediatric intensive care unit

Variables		Overall (n=135)	Boys (n=86)	Girls (n=49)	p-value
Categorization of patients	Dengue fever	92 (68.1%)	58 (67.4%)	34 (69.4%)	0.249
	Dengue with warning signs	22 (16.3%)	14 (16.3%)	8 (16.32%)	
	Severe dengue	21 (15.6%)	14 (16.3%)	7 (14,3%)	
Outcome	Recovered	131 (97.0%)	84 (97.7%)	47 (95.9%)	
	Referred to PICU	4 (2.96%)	2 (2.33%)	2 (4.08%)	0.563
	Death	0 (0%)	0 (0%)	0 (0%)	
Duration of stay in hospital, days		3.73±1.90	3.83±1.80	3.55±2.07	0.422

## Discussion

The case burden of dengue has escalated in a risky manner over the last few years [77]. The present situation of dengue in Bangladesh is also alarming, causing an extra economic burden [78]. In our study, dengue was more prevalent in boys, consistent with most studies [79-83]. However, studies from Columbia and Thailand showed female predominance [84,85], and one study observed equal distribution [86]. Like other studies, most of our dengue-affected children (n=46, 34%) belonged to the 5-10 years group [82,86-88]. The mean age was 91.6 months, comparable to other studies [80,81], although few authors differed [26,47,89,90]. Infants comprised 8.9% (n=12) of total patients. Several authors observed that most of the affected pediatric patients were more than 15 years of age [81,85,91,92]. Most affected children were from the nearby locality, belonged to lower and middle socioeconomic conditions, and had normal nutritional status, which is in line with the findings of other researchers [26,83]. In Colombian children, the percentage of overweight was a considerable portion [84], probably due to economic differences. Besides nutritional abnormalities, other comorbidities were observed in 5.9% (n=8) of patients, less than in other studies [83,92].

Similar to other studies, fever was the most frequent clinical symptom [81,82,87,89,93], followed by abdominal pain (n=81, 60%) and vomiting (n=77, 57%). Although this finding is consistent with Mishra et al. [81], other authors found abdominal pain less frequent [80,82,87,92,93] as a clinical symptom. The proportion of vomiting concorded with other studies [80,92], but it was less prevalent in earlier studies [87,93]. Typical symptoms like headache (n=38, 28.2%), body aches (n=32, 23.7%), and rash (n=16, 11.9%) were found to be less ubiquitous than in other studies [82,89,92,93]. Among respiratory symptoms, the cough was the most common (n=43, 31.9%). In contrast, other authors observed less pulmonary involvement [89,92]. Among the studied subjects of this research, 17% (n=23) suffered from diarrhea, a common finding by several authors [80,87,89,93], but the percentage was higher in young Thai children [94]. In our investigated cases, 5.93% (n=8) of patients developed various bleeding manifestations, where epistaxis was the commonest. Other authors frequently found the bleeding tendency, although proportions were higher [47,80,82,83,87]. A convulsion occurred in 2.96% (n=4) of affected children, which is consistent with the findings of Mahmood et al. [80], but other authors observed higher frequency [82,87]. Seizures due to fever are common in young children [95]. In our research, we included children below 18 years; hence, our subjects' convulsions were lower. Retro-orbital pain, a classic presentation of dengue, was not frequent in our study, although few authors found it in higher frequency [80,82]. The duration of fever was 3.44±1.17, consistent with Te et al. [83].

**Figure 2:**
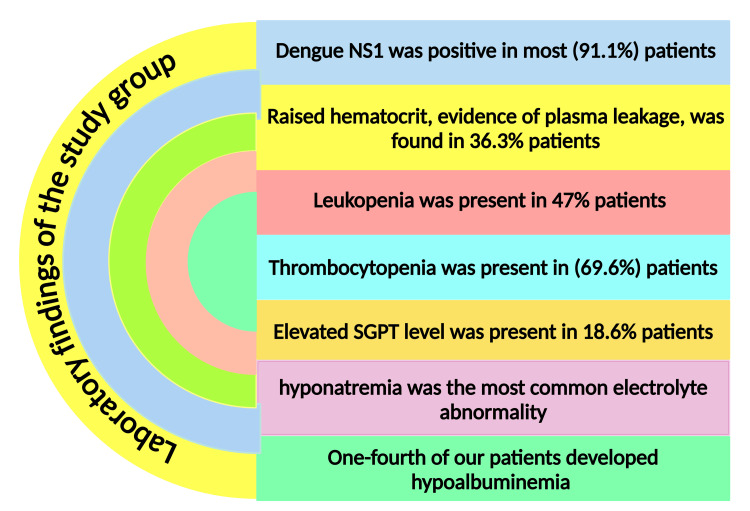
Various laboratory findings of the study group Note: The premium version of BioRender (https://biorender.com/) [76] was used to draw this figure and was accessed on November 20, 2024 with license number VQ27KJR8UH. Illustration Credit: Susmita Sinha NS1: nonstructural protein 1; SGPT: serum glutamate pyruvate transaminase

Our patients (n=16, 11.9%) had hypotension, consistent with the findings of Bodinayake et al.'s research [92]. Comparable to other studies [81,87], signs of plasma leakage in the form of ascites and/or pleural effusion were present in 26.7% (n=36) of patients. This percentage was variable in a few studies [80,82,92]. Dengue NS1 was positive in most (n=123, 91.1%) of our patients, consistent with other researchers' findings [80,81,84,89,91,96,97]. Raised hematocrit was found in 36.3% (n=49) of our patients, comparable to the study by Mishra et al. [81]. Only a tiny proportion of our patients developed reduced hematocrit due to fluid mismanagement or active bleeding, like in an earlier study [87]. Leukopenia was present in 47% of our patients, consistent with other authors [47,81,84,96]. Patients (n=94, 69.6%) developed thrombocytopenia, corresponding with other authors [87,96,97]. Platelet count was severely reduced (<20,000/cu mm) in 14% (n=19) of children, which was less observed in children from India [82] and Sri Lanka [98]. Elevated SGPT level was present in 18.6% (n=25) of patients, consistent with children from Pondichéry [82], but the proportions are higher in other studies (Figure 2) [85,87,99]. One-fourth of our patients developed hypoalbuminemia (Figure 2), comparable to other research [90], although the percentage was lower in a few studies [85,96]. Hyponatremia was the most common electrolyte abnormality. It was analogous to other studies [87].

Ascites and pleural effusion were the most frequent sonological abnormalities in other studies [81,82,92]. On chest X-ray, pleural effusion was revealed in 28.9% (n=39) of children, consistent with other authors [80,81]. Five cases (3.7%) showed hepatomegaly. It was similar to findings from Sri Lankan children [92] but much less than the other studies [81,96].

All patients utilized oral rehydration solution (ORS). When the intravenous fluid was needed, crystalloid, plasma, and albumin infusion were used in 86.1% (n=116), 8.89% (n=12), and 5.93% (n=8) of cases, respectively. The use of intravenous fluid was less in other studies [82,83,92]. Among our study participants, 8.89% (n=12) also required platelet transfusion, but none did not need whole blood transfusion, similar to the observation by Te et al. [83].

Although antibiotics are not indicated for DF, they were prescribed in 15.6% (n=21) of patients due to bacterial coinfection. The presence of antibiotics and the presence of coinfection varied in different studies [82,83,92].

Most of our patients (n=92, 68.1%) belonged to group A, similar to others [80]. Although dengue patients without warning signs (group A) are not indicated for hospital admission according to guidelines, we had to admit them due to anxiety and concern about the adequacy of fluid intake by parents or caregivers. This reflects the lack of awareness and poor counseling of the parents about this medical condition. Severe dengue was present in 15.6% (n=21) of our cases, consistent with other authors [26,81,92], but this percentage varied in several different studies [47,83,85,86].

Most patients recovered, with a median duration of hospital stay of 3.73 days, comparable to other researchers [81,83,92]. A slightly longer duration was noted by Ahmed et al. [87], probably because younger children required more time to be treated than older ones. Notably, a few patients (n=4, 2.96%) were referred to the PICU for better management, consistent with Sri Lankan children [92]. Fortunately, no mortality was reported in the present study, probably due to admission bias from the emergency room. This could also reflect the increased awareness and the availability of rapid diagnostic tests, resulting in prompt diagnosis and effective management. This finding of absolute zero mortality is consistent with other studies [87,92].

Limitations of this study

This is a descriptive study with its inherent limitations. The descriptive research plan is to portray the dispersal of one or more variables exclusive of concern to any causative or other postulation [100,101]. Bellomo et al. (2009) reported that "single-center studies frequently either lack the scientific rigor or external validity required to support widespread changes in practice, and their premature incorporation into guidelines may make the conduct of definitive studies more difficult" [102]. This paper was a single-center study, so it may not represent the general population. However, our study sample size was not too small (135) [103]; nevertheless, it was comprised of a small number of study participants, as per the context of the country (Bangladesh population size = 173,562,364) in 2024 [104].

Moreover, this study was out-of-pocket expenses, and no institutional financial support was received. Therefore, we cannot conduct a multicenter study and wait to increase the length of the study period and the sample size of hospitalized patients. Dengue cases were diagnosed based on the dengue NS1 or anti-dengue antibody tests; PCR or viral isolation was not done. Hence, patients with dengue might have been missed. The study was done for only four months, which may bias the result. No relationship between the age of the patients and clinical manifestations was sought.

Research recommendation

A well-designed multicenter prospective study with more substantial study participants is recommended to elicit the relationships/associations with more patient characteristics. 

## Conclusions

DF is a global health problem causing significant morbidity and mortality. It has become an endemic disease in many parts of the world, including Bangladesh. Our study has shown that there may be a wide variety of clinical manifestations in dengue-affected children, which may create dilemmas during clinical diagnosis. Lab tests like NS1 antigen for dengue are an effective tool for early diagnosis of dengue, which is crucial for proper management and prevention of complications. Efficacious treatment also reduces hospital stays and economic burdens. Our study has shown that most dengue patients came without warning signs and could be managed at home, but overconcern and undue anxiety compelled the parents to unnecessary hospital admission. Effective management according to national guidelines also ensures better outcomes for hospital-admitted patients.
